# Visual Grouping in Accordance With Utterance Planning Facilitates Speech Production

**DOI:** 10.3389/fpsyg.2018.00307

**Published:** 2018-03-12

**Authors:** Liming Zhao, Kevin B. Paterson, Xuejun Bai

**Affiliations:** ^1^Key Research Base of Humanities and Social Sciences of the Ministry of Education, Academy of Psychology and Behavior, Tianjin Normal University, Tianjin, China; ^2^Department of Neuroscience, Psychology and Behaviour, University of Leicester, Leicester, United Kingdom

**Keywords:** visual grouping, syntactic planning, Gestalt principles, language production, phrasal organization

## Abstract

Research on language production has focused on the process of utterance planning and involved studying the synchronization between visual gaze and the production of sentences that refer to objects in the immediate visual environment. However, it remains unclear how the visual grouping of these objects might influence this process. To shed light on this issue, the present research examined the effects of the visual grouping of objects in a visual display on utterance planning in two experiments. Participants produced utterances of the form “The snail and the necklace are above/below/on the left/right side of the toothbrush” for objects containing these referents (e.g., a snail, a necklace and a toothbrush). These objects were grouped using classic Gestalt principles of color similarity (Experiment 1) and common region (Experiment 2) so that the induced perceptual grouping was congruent or incongruent with the required phrasal organization. The results showed that speech onset latencies were shorter in congruent than incongruent conditions. The findings therefore reveal that the congruency between the visual grouping of referents and the required phrasal organization can influence speech production. Such findings suggest that, when language is produced in a visual context, speakers make use of both visual and linguistic cues to plan utterances.

## Introduction

An important issue in speech production concerns how speakers generate preverbal messages (Huettig et al., 2011). Although detailed models of utterance planning have been developed (e.g., Dell, 1986; Levelt, 1989; Levelt et al., 1999; Indefrey and Levelt, 2004), little is known about how these processes coordinate with non-linguistic information. For instance, in tasks where language is produced in a visual context, such as giving directions from a map, visual and linguistic information are thought to be synchronized and to draw upon cross-modal cognitive mechanisms that allow different modalities to share, exchange, and integrate information (Coco and Keller, 2015). However, few studies have examined the influence of the structure of information in the visual context on utterance planning. Accordingly, to shed light on this issue, the present study assessed on the interplay between visual information and utterance planning during language production.

A promising line of inquiry comes from research that has adapted the visual world paradigm to study language production and shows a strong link between the direction of gaze and speech planning (for a review, see Huettig et al., 2011). On one hand, studies show that speakers strongly prefer to look at the objects they refer to and visually attend to those objects in their order of mention (e.g., Meyer et al., 1998; Griffin and Bock, 2000; Griffin, 2001), revealing an influence of word order on visual processing. On the other hand, other research shows a contrary pattern of behavior in which visual processing determines word order in language production (Brown-Schmidt and Tanenhaus, 2006; Gleitman et al., 2007). For instance, Gleitman et al. asked participants to describe events unfolding in a cartoon. Just prior to the onset of each display, a brief visual cue appeared in the position of one characters. Although participants were unware of the cue, they nevertheless were more likely to direct initial fixations toward the cued character and to mention this character earlier in an utterance than an uncued character. This effect was taken to show that the visual cue captured the speaker's visual attention and that directing attention to the cued character facilitated the retrieval of the character's name, which in turn increased the likelihood of this character being mentioned early in an utterance.

These previous studies have focused on the relationship between the order of gaze on referents and their order of mention in utterances. However, visual information is often more highly structured and it is unclear if only order of gaze is important in influencing the order of mention of referents in utterances. To further explore the influence of visual structure, Bock et al. (2003) examined the integration of visual and linguistic information in a time-telling task. Speakers were presented with analog and digital clock displays and asked to tell the time using either absolute expressions (e.g., “two fifty”) or relative expressions (e.g., “ten to three”). Bock et al. assumed that analog displays would be more compatible with relative expressions, and digital ones with absolute expressions, and therefore that the design would enable a comparison of situations in which the required linguistic form was more or less compatible with the display. Consistent with this logic, they found compatibility effects in the onset latency of utterances for both absolute and relative expressions and took this to reveal an interaction between visual and syntactic information beyond that of processing order. However, the visual representations they used are unique and, moreover, the differences between relative and absolute expressions still concerned word order (whether hour or minute information was produced first). Consequently, further work is needed to demonstrate the generality of these observations.

**Figure 1:**
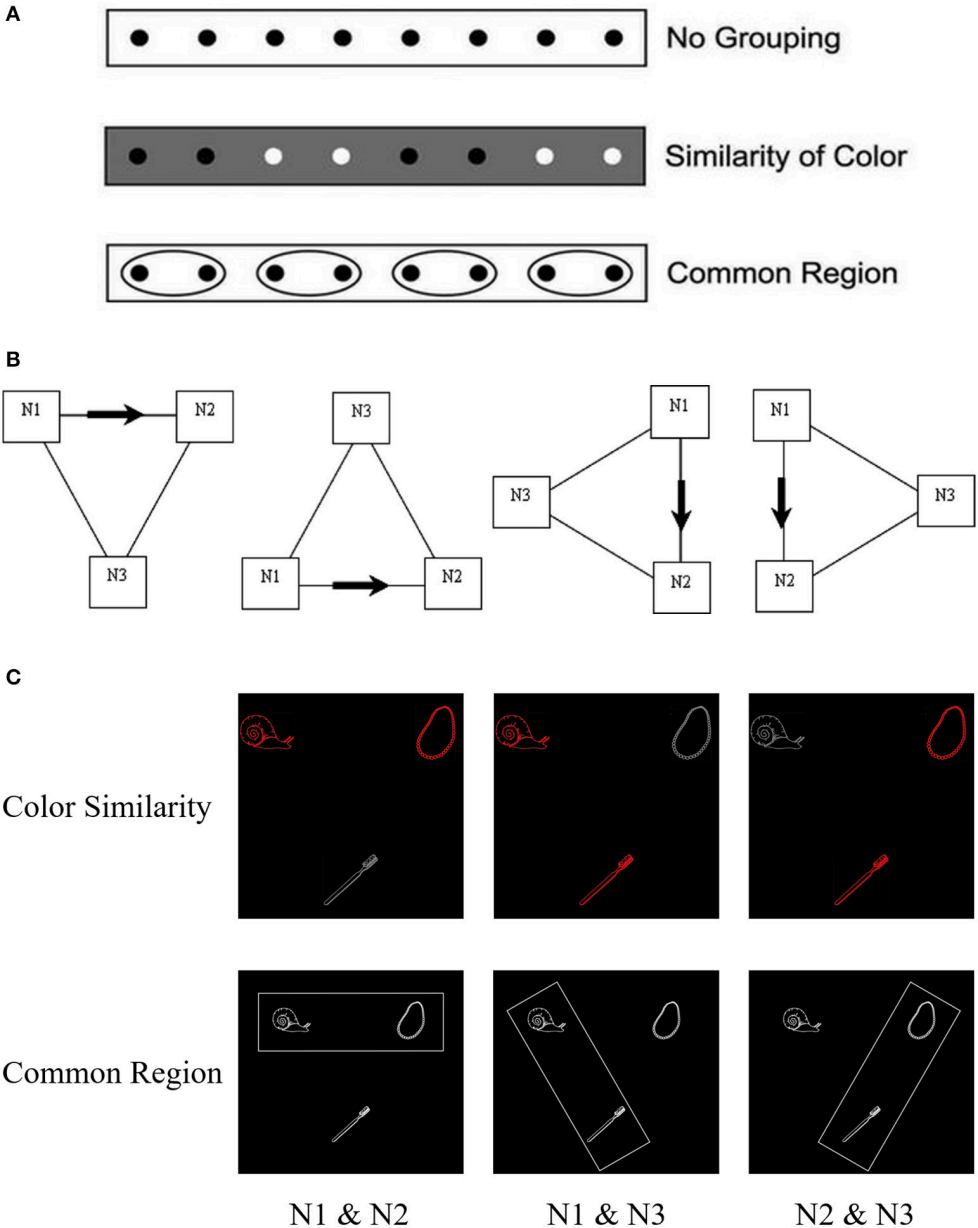
Examples of **(A)** grouping principles, **(B)** the spatial configuration of objects in displays, and **(C)** examples of displays used in Experiments 1 and 2. **(A)** Illustration of grouping principles as similarity of color and common region (adapted from Palmer, 2002). **(B)** The four kinds of location relationships among the three pictures: above, below, right, and left (The arrows indicate the directions of description. Neither of the boxes, arrows, or lines was provided as part of the visual array. See the same presentation in Yang and Yang, 2008). **(C)** Example displays in the “above” location to be described with the sentence “the snail and the necklace are above the toothbrush” in the three grouping conditions of color similarity (Experiment 1) and common region (Experiment 2).

The present research aimed at providing a more general indication that this influence of the visual organization of information extends beyond cueing the order of mention of referents. The approach made use of Gestalt principles of perceptual organization introduced by Wertheimer (1923/1938) and further developed by Köhler (1929). These specify principles underlying visual grouping, including grouping based on color similarity or common region (Wagemans et al., 2012). The principle of color similarity stipulates that similarly-colored objects tend to be grouped, while the principle of common region requires that elements that lie within the same bounded area tend to be grouped (see Figure 1A). We used these principles to examine effects of visual grouping on utterance planning. Specifically, if referents are grouped by color-similarity or common region this may influence how phrases are organized so that utterance planning is facilitated when visual grouping and phrasal organization are congruent rather than incongruent.

To test this proposal, we conducted two experiments in which color-similarity (Experiment 1) or common region (Experiment 2) was manipulated and participants produced utterances of the form “The snail and the necklace are above/below/on the left/right side of the toothbrush” for displays containing three referents (e.g., a snail, a necklace and a toothbrush; see Figure 1B). In Experiment 1, the influence of color-similarity was examined for three configurations in which objects corresponding to the first and second noun (N1 & N2), first and third noun (N1 & N3), or second and third noun (N2 & N3) were of the same color and the other object had a different color. In Experiment 2 a rectangle was used to bound objects corresponding to two of the nouns within a common region while excluding the third to produce the same configuration of visual groupings as Experiment 1.

We expected a congruency effect if speakers integrate these visual grouping cues with syntactic planning when preparing an utterance so that speech onset latencies are shorter in congruent than incongruent conditions. However, performance may also be influenced by a further perceptual factor identified by Martin et al. (2010), which requires that objects with “common fate” (e.g., the same color or direction of movement) tend to be grouped and that this will slow lexical retrieval for any one of these grouped items. If correct, this may result in slower lexical retrieval for N1 when N1 is grouped with N2 or N3 in the present experiments.

Various studies show such effects on lexical access (e.g., Smith and Wheeldon, 1999; Allum and Wheeldon, 2007; Martin et al., 2010; Zhao et al., 2015). However, this is unlikely to confound our findings in the present research, for two reasons. First, while such effects have been found for color-based grouping (Zhao et al., 2015), they are not found for spatially-grouped objects (Martin et al., 2010). Consequently, although this perceptual influence may affect performance in Experiment 1 when referents are grouped by color, such an effect is unlikely in Experiment 2 when referents are grouped by common region. Second, because the grouping of the referents was varied systematically in the present experiments, such an effect is equally likely in congruent and incongruent conditions, and so unlikely to confound our findings. Accordingly, regardless of this influence of visual grouping on lexical retrieval, we should observe congruency effects due to color grouping in Experiment 1, and spatial grouping in Experiment 2, if speakers integrate visual cues during syntactic planning when preparing utterances.

## Experiment 1

### Methods

#### Participants

Participants were 25 native Chinese speakers (aged 20–25 years) from universities near the Institute of Psychology at the Chinese Academy of Sciences in Beijing. All reported normal or corrected-to-normal vision and were paid a small sum for participation. This study was approved by the Ethics Committee of the Institute of Psychology, Chinese Academy of Sciences in Beijing, and conducted in accordance with the ethical principles of the Declaration of Helsinki.

#### Stimuli and design

The experiment used 30 pictures of objects from the Snodgrass and Vanderwart database (Snodgrass and Vanderwart, 1980) that had two-character names in Mandarin Chinese. These were divided into 3 groups, matched for naming latency, using norms from Zhang and Yang (2003). They were then arranged into sets of three by selecting one picture from each group pseudo-randomly so that pictures in each set were semantically-unrelated, had phonologically-unrelated names (in Chinese), and looked dissimilar. This produced 10 triplets/items (see Appendix). For each triplet, 12 display permutations were created by varying combinations of object color and spatial arrangement (3 color × 4 spatial arrangements; see Figures 1B,C). In addition to the experimental items, 12 warm-up trials and 24 practice trials (used in a familiarization session prior to the experiment) were created following the same procedures. The GPower (3.1) application was used to check the power of our study (Faul et al., 2007). Setting α at 0.05, 1-β at 0.8, and the effect size *f* at a low level of 0.1, the estimation showed that the total sample size needed was *n* = 969. This indicated that the sample size in this study of *n* = 3,000 (25 subjects ^*^ 10 items ^*^ 12 display permutations) was sufficient to achieve a power of at least 80%.

In Experiment 1, color-similarity was manipulated so that objects corresponding to either the first and second noun (N1 & N2), first and third noun (N1 & N3), or second and third noun (N2 & N3) had the same color and the other object had a different color. The primary dependent variable was the onset latency of utterances, although we also examined errors in which participants used unexpected content words, incorrect syntax, or produced a disfluency (repair, stutter, hesitation, or nonverbal sound) that would trigger the voice key inappropriately.

The object pictures were scaled to fit 175 × 175 pixels frames. These were shown in red or gray on a black background, and luminance was matched across color conditions in Experiment 1. In each display, each set of 3 pictures was presented at the vertices of a virtual equilateral triangle whose center was at the middle of the display. The distance between any 2 objects (center to center) was 525 pixels. Stimuli were presented in 4 blocks, each comprising 30 experimental trials with 3 warm-up trials at the beginning of each block. Each experimental triplet was shown 3 times per block, in each color combination, and the spatial arrangement of displays was counterbalanced across blocks. Within each block, trials were presented in pseudo-random order so that trials involving the same objects, color combination, or spatial arrangement did not appear consecutively. The order of blocks was rotated across participants.

#### Procedure

Participants were tested individually and received written instructions. Before the experiment, participants took part in a familiarization session in which they saw the object pictures paired with their names. Participants were then seated 70 cm from a display monitor. They first completed a practice session consisting of 24 trials counterbalanced for color and spatial arrangement, before starting the experiment. In the instruction for the practice session, participants were shown with the Figure 1B to illustrate the four types of spatial arrangements and instructed as “Welcome to the study! At the beginning, there will be a ‘+’ in the center of the screen. Please focus on the ‘+’. Then the ‘+’ disappears and three objects will be presented as one of the four spatial arrangements as presented in the figure. Please prepare a sentence as ‘the N1 and the N2 are above/below/on the left/right side of the N3,' and produce it as accurately and soon as possible. In the figure the arrows indicate the directions of description. Neither of the boxes, arrows, or lines was provided as part of the visual array. The N1, N2, and N3 should be changed to the corresponding names of the objects.” During the practice, we would correct participant's responds if they used unexpected names or syntactic structures, to make sure they understand and get used to the instruction before the formal experiment. At the beginning of each trial, a fixation point was shown for 1,000 ms at the screen center, followed by a stimulus presentation for 4,000 ms. Participants were instructed to produce an utterance of the required form as quickly and accurately as possible. Trials were separated by a 2,000 ms interval during which the display was blank. Participants were given a short break between sessions and blocks. The experiment lasted approximately 40 min for each participant.

### Results

In this and a subsequent experiment, we report the analyses of correct RT and error rates for the fixed factor of color similarity, using linear mixed effects model with subjects and items as crossed random factors (Baayen, 2008). The dependent variables were speech onset latency and error rate. The items referred to the sets each consisting of three pictures (see Appendix).

Of the 120 experimental trials, recording failures and no response made within the 4,000-ms timeout period were excluded from the analyses. Then we examined the shape of the RT distribution, and excluded the data points faster than 200 ms and longer than 3,000 ms as outliers to meet the distributional assumption of the linear mixed effects model. All the excluded trials accounted for 1.3% of the data.

Production errors were scored as using unexpected content words, using incorrect syntax, and fluency problems (repairing, stuttering, hesitation, and production of nonverbal sounds that triggered the voice key). Such trials accounted for 11.5% of the data and were excluded from the correct RT analyses. The correct mean RTs and error rates for the three levels of color similarity are summarized in Table 1.

**Table 1:** Mean latencies and percentage error rates for two experiments (participant means).

	**Grouping condition**	**Latency (ms)**		**Errors (%)**
		**Mean**	***SD***	
Experiment 1 (color similarity)	N1 & N2	1,222	338	11.1
	N1 & N3	1,261	341	12.5
	N2 & N3	1,266	358	11.3
Experiment 2 (common region)	N1 & N2	1,181	286	5.2
	N1 & N3	1,271	305	6.5
	N2 & N3	1,316	311	5.4

#### Correct RT

The data were submitted to a linear mixed effects model using the lme4 package (Bates et al., 2013, Version 1.1–5) implemented in R 3.0.3 (R Core Team, 2014). Degrees of freedom (estimated using Satterthwaite's approximation) and *p*-values were estimated using the lmerTest package (Kuznetsova et al., 2013, Version 2.0–11). In line with the recommendation to keep the random effect structure maximal (Barr et al., 2013), the initial model included random slopes on color similarity, but did not converge. The final model we report included only the subject and item intercepts. Using R syntax, the model was: RT ~ color-similarity + (1 | subject) + (1 | item), with 25 subjects and 10 items. The contr.treatment in R was used to compute contrasts. For our study purpose, we firstly used the N1 & N2 condition (congruent condition) as the reference and the two incongruent conditions as the contrast. The model's estimates of the effect of each color-similarity condition, the associated standard error, estimated degrees of freedom, and *t* and *p*-values are shown in Table 2.

**Table 2:** The model's estimate, standard error (std. error), degrees of freedom (*df*), *t*-value, and *p*-values of fixed effects for the correct RT in Experiments 1 and 2.

	**Grouping condition**	**Estimate**	**Std. error**	***df***	***t*-value**	**Pr(>|*t*|)**
Experiment 1 (color similarity)	(Intercept)	1224.31	44.63	28.5	28.059	<0.001
	N1 & N3	40.23	13.42	2598.2	2.998	<0.01
	N2 & N3	41.54	13.35	2598.3	3.111	<0.01
Experiment 2 (common region)	(Intercept)	1179.38	33.93	33.5	34.758	<0.001
	N1 & N3	90.38	12.04	2778.7	7.509	<0.001
	N2 & N3	133.94	12.02	2779.8	11.147	<0.001

The model showed that referenced to the congruent condition in which N1 and N2 were in the same color, speakers spent more time to prepare the utterances in the other two incongruent conditions: N1 & N3, *t* = 2.998, *p* < 0.01; N2 & N3, *t* = 3.111, *p* < 0.01. In addition, we changed the N1 & N3 condition as the reference and the other two conditions as the contrast, and found that there was no significant difference between the two incongruent conditions (*t* = 0.098). We had performed analyses using logRTs as well, and it produced the same pattern of results.

#### Error rate

The error data were analyzed using a logit mixed model (Jaeger, 2008) using the same model as for correct RT. The model showed that there were no significant differences among the three conditions of color similarity (*z*s < 1).

### Discussion

The findings showed that speech onset latencies were shorter when the visual grouping of objects was congruent with their phrasal organization in utterances. The findings therefore suggest that visual and linguistic information is integrated during utterance planning. Our findings are in line with the compatibility between visual context and the required linguistic form reported by Bock et al. (2003) but show that this effect generalizes to include the visual grouping of objects based on color.

The findings do not exclude the possibility that perceptual grouping slowed lexical retrieval of object names (Martin et al., 2010; see also Zhao et al., 2015), although this would have occurred with equal likelihood across the different conditions and so independently of the manipulation of perceptual grouping in the present experiment. It was nevertheless valuable to determine if the same effect of visual grouping on utterance planning is observed when this visual interference with lexical retrieval is not predicted. Therefore, in Experiment 2 we further assessed the influence of perceptual organization on utterance planning by manipulating the grouping of object referents in terms of common region.

## Experiment 2

### Methods

#### Participants

Participants were 25 native Chinese speakers (aged 19–23 years) from Tianjin Normal University.

#### Materials, design, and procedure

Experiment 2 used the same materials, design, and procedure as Experiment 1, except that all object pictures were the same color (white) and luminance, and a rectangular frame was used to indicate a common region. The rectangular frame was always the same color (white) and size (700 × 220 pixels) but rotated 60° to produce 3 common region (see Figure 1C) so that the rectangle bounded objects corresponding to the first two nouns (N1 & N2), first and third nouns (N1 & N3), or second and third nouns (N2 & N3).

### Results

Data were excluded using the same criteria as Experiment 1. All the excluded trials as outliers accounted for 0.1% of the data. Error trials accounted for 5.7% of the data. The correct mean RTs and error rates for the three levels of common region are also summarized in Table 1.

#### Correct RT

The same model was used to analyze correct RT for the fixed factor of common region, which in R syntax was: RT ~ common-region + (1 | subject) + (1 | item), with 25 subjects and 10 items. The common-region factor was referenced to the N1 & N2 condition. The model's estimates of the effect of each common-region condition, the associated standard error, estimated degrees of freedom, and *t* and *p*-values are shown in Table 2.

The model showed that referenced to the congruent condition in which N1 and N2 were in the same contour, speakers spent more time to prepare the utterances in the other two incongruent conditions: N1 & N3, *t* = 7.509, *p* < 0.001; N2 & N3, *t* = 11.147, *p* < 0.001. In addition, we changed the N1 & N3 condition as the reference and the other two conditions as the contrast, and found that the onset latencies in the N1 & N3 condition were significantly faster than in the N2 & N3 condition (*t* = 3.614, *p* < 0.001). As in Experiment 1, we performed analyses using logRTs as well, and it produced the same pattern of results too.

#### Error rate

The error data were analyzed similarly as in Experiment 1, using the same model as for correct RT. The model showed that there were no significant differences among the three conditions of common-region (*z*s < 1.3).

### Discussion

Speech onset latencies were shortest when the visual grouping was congruent with syntactic planning, replicating the congruency effect in Experiment 1. Experiment 2 therefore provided further evidence that the congruency of visual grouping with syntactic planning facilitates speech production, and therefore that visual grouping and syntactic planning interact during speech production. The findings also show that this effect generalizes to visual grouping based on common region.

However, in Experiment 2 we observed a difference between the incongruent conditions which was not observed in Experiment 1 when visual grouping was specified in terms of color similarity. This showed that onset latencies were shorter when the first-produced noun was inside rather than outside the common region. This effect does not undermine the congruency effect we observed, but suggests additional factors may influence utterance planning. This unexpected difference in onset latencies for the incongruent conditions, in particular, may reveal an effect of visual attention. Accounts predict that objects close to a contour, such as those within the rectangle in our experiment, receive more attention than objects further from the contour, such as those outside the rectangle (Arnay and Acosta, 2014; Pooresmaeili and Roelfsema, 2014) and that this can facilitate the processing of these objects. Accordingly, more attention to N1 when it is bounded by the rectangle may speed N1 processing (by facilitating both recognition and lexical retrieval) and so produce shorter onset latencies, and this may explain why onset latencies were shorter for incongruent conditions when N1 was bounded than when not. This interpretation is based on the assumption that the N1 must be accessed before speech onset and this is reflected in the speech onset latencies, which has been confirmed by many studies (e.g., Griffin, 2001; Zhao and Yang, 2016). However, this will not conflict with the rationale and conclusion of this study, because the difficulty in accessing N1 would be equivalent between N1 & N2 (congruent) and N1 & N3 (incongruent) conditions. In these two conditions, the N1 was both bounded by the rectangle. The only difference between these two conditions was whether the visual grouping of common region was congruent with the phrasal organization. Thus the difference in speech onset latencies between N1 & N2 and N1 & N3 conditions is still attributed to the interaction between visual grouping and syntactic planning rather than lexical access.

## General discussion

Two experiments provide clear evidence that the congruency between the visual grouping of referents in a display and the organization of phrases during syntactic planning can influence speech production. In Experiment 1 speech onset latencies were faster when objects corresponding to nouns in the same complex noun-phrase (e.g., “the snail and the necklace”) were the same rather than a different color. In addition, Experiment 2 showed the similar facilitation when objects corresponding to this noun-phrase were within the same bounded region than when not.

These findings are consistent with previous research showing rapid integration of visual and linguistic information during speech production. Indeed, many studies show that the order of words in utterances formats influence speaker's gaze patterns (e.g., Meyer et al., 1998; Griffin and Bock, 2000; Griffin, 2001), while other studies show that the order in which objects are visually inspected can affect the order in which words are produced in utterances (Bock et al., 2003; Brown-Schmidt and Tanenhaus, 2006; Gleitman et al., 2007). Crucially, the present findings show that this interplay between visual processing and speech planning generalizes beyond effects of word order by revealing that the visual grouping of objects in terms of color similarity or common region can also influence syntactic planning.

Such findings resonate closely with other observations that visual context has a rapid mediating influence on syntactic planning during spoken language comprehension (e.g., Tanenhaus et al., 1995). These findings may also be relevant to more general observations that a close relationship exists between language understanding and perceptual/motor processes. For example, Glenberg and Kaschak (2002) found that when a sentence implied action in one direction (e.g., “Close the drawer” implies action away from the body), participants had difficulty enacting a judgment that required making a physical response in the opposite direction. This and similar studies suggests that comprehension involves the perceptual or motor mental simulation of described events, and therefore a close yoking between language and perception. However, while many studies use visual world paradigms in which researchers investigate the influence of presenting specific objects in a visual display on the nature and timing of speech production, the present study is one of the first to examine the influence of the structure of this visual information on utterance planning. Our findings show that processes of speech production also naturally recruit information from perceptual processes, and this may provide a basis for further investigations of the relationship between perception and language production.

In the present study, the visual grouping of objects according to their color-similarity or common-region was manipulated to be congruent or incongruent with the phrasal organization. The results indicate that speech production was slower when the visual grouping and utterance planning were in conflict. However, one objection to these findings could be that the visual grouping of the objects interfered with the ability to establish the spatial relationship between the object (e.g., that the snail and the bracelet are above the necklace) rather than the utterance planning required to express this relationship. This is difficult to disentangle. However, it should be noted that the spatial relations between the objects was always unambiguous and it is unclear how grouping these objects in terms of either color or common region would be likely to interfere with the computation of quite simple spatial relations. For this reason, we consider this alternative explanation of our results to be unlikely.

Various studies show the interference effect of visual grouping on the object identification. That is, better separation of the alternative possible goals can help people select the intended one faster and more correctly (e.g., Chen and Proctor, 2014). Accordingly, it is more difficult to recognize the object and retrieve its corresponding noun when the intended object is visually grouped with other objects (Zhao et al., 2015). However, this is unlikely to confound our findings in the present research, for two reasons. First, for the identification of the three objects in each trial, because the grouping of the referents was varied systematically in the present experiments, such an effect is equally likely in congruent and incongruent conditions, and so unlikely to confound our findings. Second, even though only the N1 is accessed before speech onset and reflected in the speech onset latencies (e.g., Griffin, 2001; Zhao and Yang, 2016), this will not conflict with the conclusion of this study, because the N1 referent was visually grouped with another object both in the N1 & N2 (congruent) condition and the N1 & N3 (incongruent) condition. The only difference between these two conditions was whether the visual grouping of color-similarity/common-region was congruent with the phrasal organization. Therefore, the difference in speech onset latencies between N1 & N2 and N1 & N3 conditions is still attributed to the interaction between visual grouping and syntactic planning rather than object identification/lexical access.

Finally, an unexpected aspect of the findings from Experiment 2 highlighted the additional role of attention in the interplay between visual processing and utterance planning. The findings showed that, for incongruent conditions, speech onset latencies were shorter when the first noun was contained within the region bounded by the rectangle than not. We attributed this effect to greater visual attention on the first noun when it is surrounded by a contour (i.e., within the rectangle; e.g., Arnay and Acosta, 2014; Pooresmaeili and Roelfsema, 2014), thereby facilitating lexical retrieval. This effect is also in line with other observations that visual cues that attract a speaker's attention to specific referent in a display can facilitate the retrieval of that referent's name, and also increase the likelihood of this referent being mentioned early in the utterance (Gleitman et al., 2007). The indication, therefore, is that both structure and the allocation of attention within a visual display can influence the planning of utterances and so highlights the importance of considering both factors in future research.

In sum, the present results provide clear evidence for an interaction between visual grouping and syntactic planning in tasks in which language is produced in a visual context. The indication from these present is that syntactic planning is sensitive to the visual grouping of referents in terms of both color similarity and common region, although the findings also show that visual attention may be an important mediator. Such findings suggest that language production may naturally recruit information from perceptual systems to help specify syntax relationships between referents during utterance planning.

## Author contributions

LZ, XB: conceived and designed the experiments; LZ: performed the experiments; LZ: analyzed the data; LZ: contributed reagents, materials, analysis tools; LZ, KP: wrote the paper.

### Conflict of interest statement

The authors declare that the research was conducted in the absence of any commercial or financial relationships that could be construed as a potential conflict of interest.

## Appendix

Experimental pictures used in the two experiments.

**Table:**

**Item**	**Object**	**Pronunciation**	**English**	**Item**	**Object**	**Pronunciation**	**English**
Item1	篮子	/lan zi/	Basket	Item2	萝卜	/luo bo/	Carrot
	电脑	/dian nao/	Computer		太阳	/tai yang/	Sun
	风筝	/feng zheng/	Kite		手铐	/shou kao/	Handcuffs
Item3	钢琴	/gang qin/	Piano	Item4	葡萄	/pu tao/	Grapes
	螃蟹	/pang xie/	Crab		蜡烛	/la zhu/	Candle
	蛋糕	/dan gao/	Cake		梯子	/ti zi/	Ladder
Item5	书包	/shu bao/	Pocketbook	Item6	蜗牛	/wo niu/	Snail
	烟斗	/yan dou/	Pipe		项链	/xiang lian/	Necklace
	抽屉	/chou ti/	Drawer		牙刷	/ya shua/	Toothbrush
Item7	拇指	/mu zhi/	Thumb	Item8	雪人	/xue ren/	Snowman
	喷泉	/pen quan/	Fountain		大炮	/da pao/	Cannon
	松鼠	/song shu/	Squirrel		叉子	/cha zi/	Fork
Item9	头盔	/tou kui/	Football helmet	Item10	镰刀	/lian dao/	Hook
	气球	/qi qiu/	Balloon		冰箱	/bing xiang/	Refrigerator
